# Mesenteric Fibromatosis Presenting As Acute Abdominal Pain Mimicking a Perforated Viscus: A Case Report

**DOI:** 10.7759/cureus.93142

**Published:** 2025-09-24

**Authors:** Konstantina Kostara, Meric Tez, Brandon Velazquez, Tyler Liguori, Vasileios Kostaras

**Affiliations:** 1 General Surgery, CarePoint Health, Bayonne, USA; 2 Pathology, Hudson Regional Hospital, Secaucus, USA; 3 General Surgery, Hudson Regional Hospital, Secaucus, USA

**Keywords:** desmoid tumor, fibroblastic, mesenteric fibromatosis, mesenteric tumor, soft tissue tumor

## Abstract

Mesenteric fibromatosis, formerly known as a desmoid tumor, is a rare tumor with aggressive local invasion. Most of these tumors present as painless, slow-growing masses, becoming symptomatic by means of mass effect as the tumor enlarges. Radiographic findings are nonspecific, and the diagnosis can only be established by histological evaluation. In this case report, we present a unique case of mesenteric fibromatosis in a 70-year-old male who presented for acute abdominal pain with image findings of a localized fluid collection between loops of small bowel. This unexpected presentation of mesenteric fibromatosis masquerading as a perforated viscus is surgically significant, and by documenting this case, we hope to add to the literature on this already challenging and rare pathology.

## Introduction

Mesenteric fibromatosis is a rare tumor with no potential for metastasis but aggressive local invasion. These tumors are sporadic, with an estimated incidence of two to four per million [1]. There is a wide distribution in the age of presentation, ranging from 15 to 60 years old, with a slight predominance in female patients [2]. There is a strong association with familial adenomatous polyposis (FAP) syndrome, with 5-15% of all desmoids arising in association with the syndrome [3]. The pathogenesis is incompletely understood, but emerging evidence suggests that dysregulation in the wound healing process and the Wnt/beta-catenin pathway may be involved [4]. There has also been a reported association with high estrogen states and antecedent trauma, both of which may contribute to the increased incidence of desmoid tumors in pregnancy [5,6].

Most desmoid tumors present as painless, slow-growing masses. Symptoms, if they arise, are mostly secondary to mass effect consequences of the tumor as it enlarges. Desmoid tumors can develop at various sites, but the three main locations described are the trunk/extremities, abdominal wall, and intra-abdominal. They can be multifocal at one site, but are rarely found at multiple sites in one patient [6]. Intra-abdominal desmoids, particularly mesenteric fibromatosis, may occasionally present with acute abdominal pain and radiologic findings that mimic other emergent conditions, such as small bowel obstruction or intra-abdominal abscess, which can lead to initial diagnostic confusion. Radiographic findings are non-specific, and there are no defined characteristics to distinguish desmoids from malignant soft tissue tumors. CT findings could demonstrate a well-circumscribed soft tissue mass arising from the mesentery, while on MRI, the tumors appear hyperintense on T2-weighted images [7]. However, the diagnosis of a desmoid tumor can only be established by histological evaluation [8]. Management is individualized, ranging from active surveillance for stable or asymptomatic lesions to surgical resection in selected cases, or systemic therapies such as nonsteroidal anti-inflammatory drugs, anti-estrogen agents, tyrosine kinase inhibitors, or chemotherapy for unresectable or progressive disease.

This case was notable for the striking disparity between preoperative imaging and operative findings. CT demonstrated a 10 × 6 cm fluid collection between small bowel loops, suggestive of a perforated viscus with evolving intra-abdominal abscess. Intraoperatively, however, a well-circumscribed mesenteric tumor measuring 10 × 8 × 5 cm was identified. This marked divergence highlights the diagnostic challenge of mesenteric fibromatosis, particularly when it mimics other acute abdominal emergencies.

## Case presentation

The patient was a 70-year-old male who presented to the emergency department (ED) with an acute onset of diffuse abdominal pain. The patient denied any associated obstructive symptoms, such as nausea, vomiting, constipation, or obstipation. He denied hematochezia, changes in stool caliber, fevers, chills, or weight loss. On arrival to the ED, he was found to be mildly tachycardic with systolic blood pressures in the low 100s, and had physical exam findings of abdominal distention and tenderness around the umbilicus without signs of peritonitis. He had mild leukocytosis, with a count of 11,000 cells/μL, and his chemistry results were unremarkable. A CT scan was performed, which revealed a localized fluid collection between small bowel loops, measuring 10 x 6 cm. The findings were concerning for a perforated viscus with an organized collection developing between small bowel loops. General surgery was consulted for an acute abdomen, and the patient was taken to the operating room.

**Figure 1:**
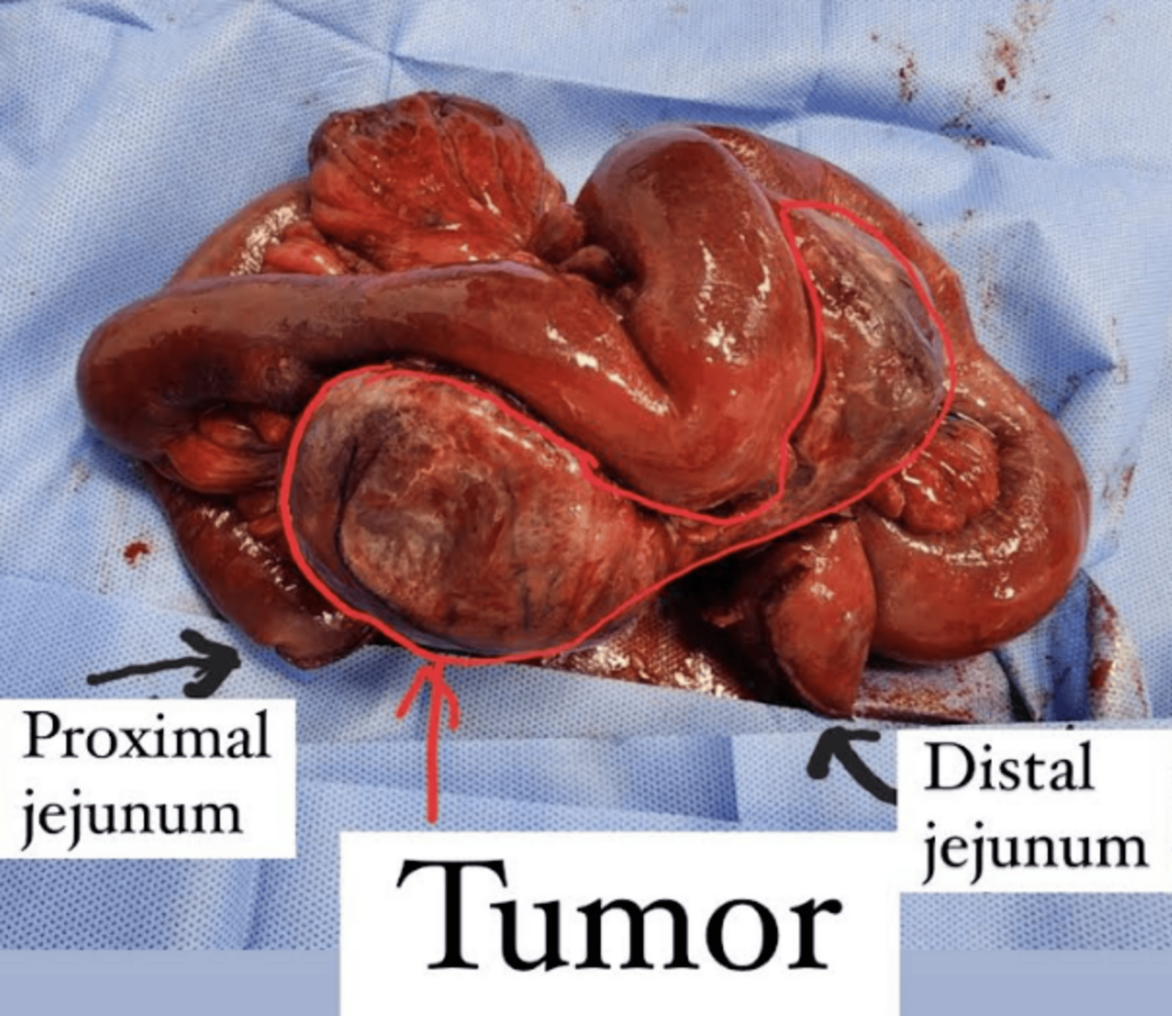
Bilobed mass measuring 10.0 × 8.0 × 5.0 cm involving the mesentery and bowel wall.

Intraoperative findings yielded a mesenteric tumor measuring 10 × 8 × 5 cm (Figure 1). The mass appeared to be invading into the proximal jejunum and abutting the superior mesenteric artery and vein. The involved mesentery was divided distally with the gastrointestinal anastomosis (GIA) stapler up to the point of healthy, soft, pliable mesentery. The involved proximal jejunum was measured at roughly 30 cm and was divided at 10 cm distal to the ligament of Treitz. En bloc resection was completed by careful dissection of the mesentery from the superior mesenteric artery and vein. A side-to-side functional end-to-end jejunojejunostomy was then performed using a blue load on the GIA stapler. The mesenteric defect was closed with 2-0 silk sutures; the remainder of the abdominal cavity was inspected with no additional abnormalities noted, and the abdomen was closed. The specimen was sent to pathology.

Pathology

Histologically, mesenteric fibromatosis consists of spindle-shaped fibroblastic cells arranged in fascicles within a dense collagenous stroma [9]. The tumor lacks cellular atypia and has low mitotic activity, distinguishing it from other malignant soft tissue tumors, such as gastrointestinal stromal tumors (GISTs). An aberrant Wnt/β-catenin signaling pathway leads to uncontrolled fibroblastic proliferation. Immunohistochemically, the tumor stained positive for β-catenin, which is the key diagnostic marker. Other markers that help with differential diagnosis are smooth muscle actin (SMA), vimentin, and desmin, which can be variably expressed. Markers such as DOG1, CD117, and S100 are negative, which differentiates from GIST and neural tumors [9].

**Figure 2:**
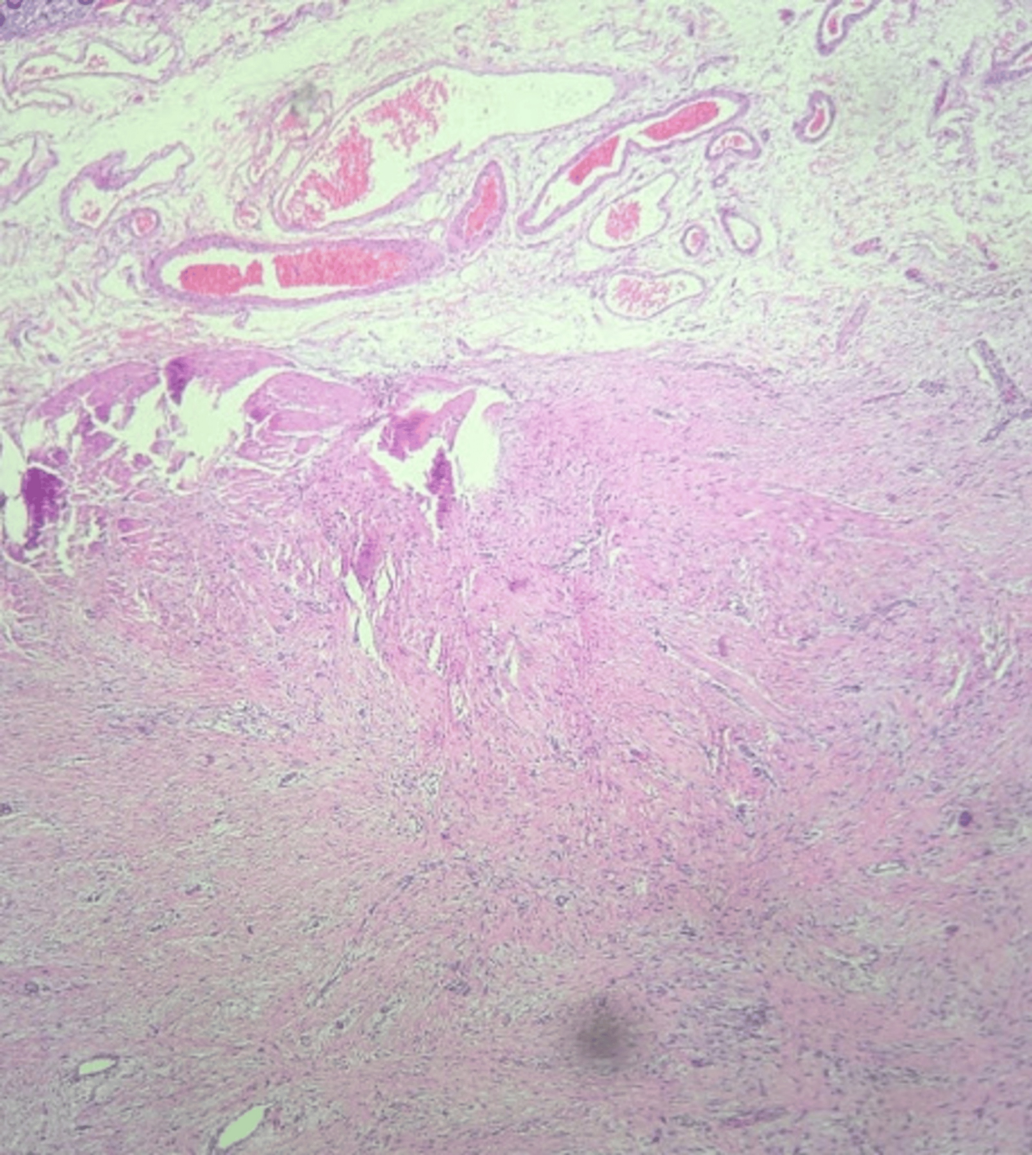
Tumor at 4x magnification showing a highly cellular, densely collagenous stroma.

**Figure 3:**
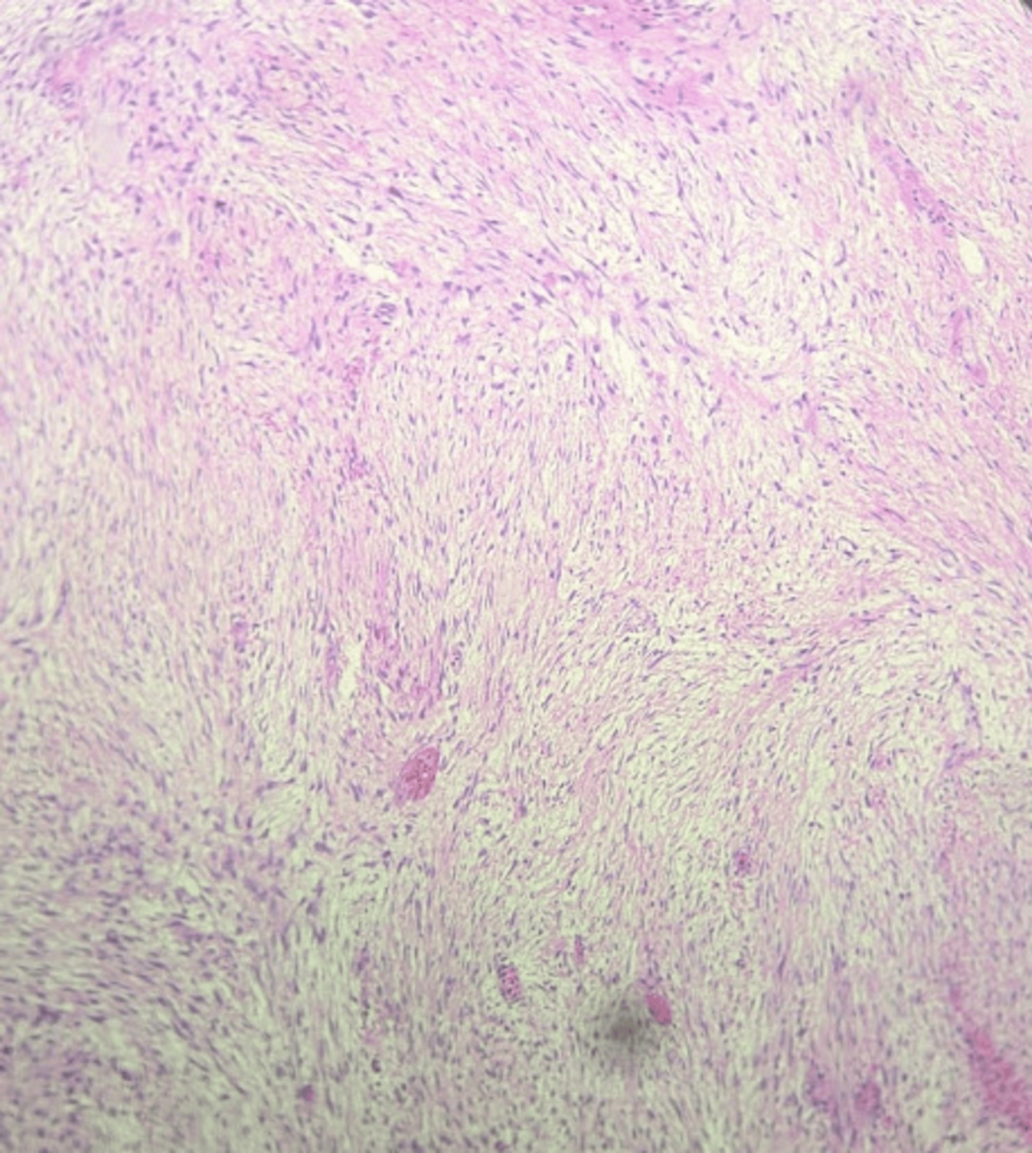
Tumor at 10x magnification showing a highly cellular, densely collagenous stroma.

**Figure 4:**
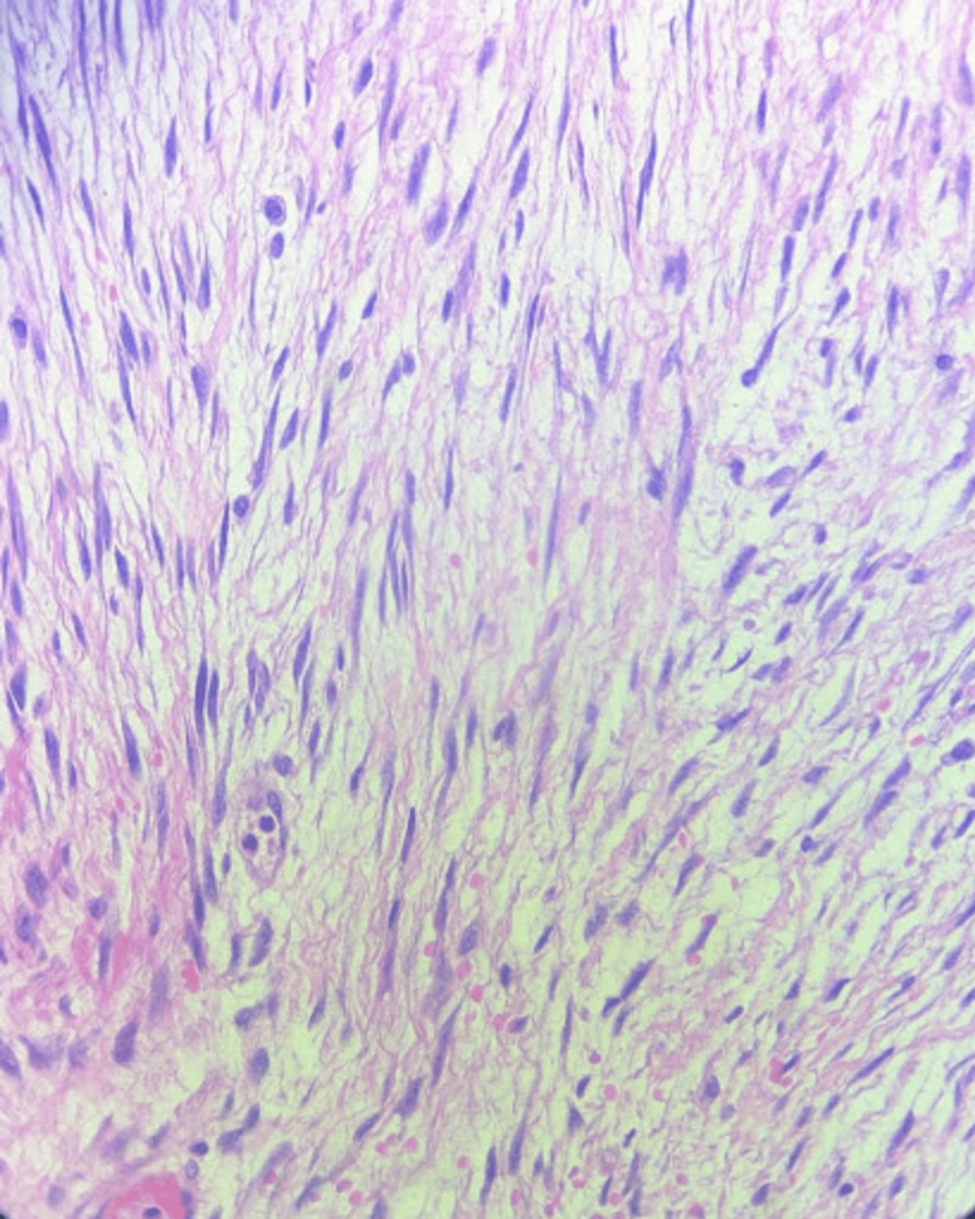
Tumor at 40x magnification showing spindle-shaped cells arranged in fascicles with low mitotic activity and cellular atypia.

Grossly, the tumor was a smooth-surfaced, firm, tan bilobed mass involving the mesentery without invading the bowel wall, as is characteristic of mesenteric fibromatosis. Cut sections showed mostly pale tan glistening surfaces with occasional fibrotic areas. No areas of necrosis or hemorrhaging were found. Initial microscopic evaluation of this specimen showed a spindle cell neoplasm involving the mesentery and small bowel wall with low-to-moderate cellularity and a dense collagenous stroma (Figures 2-4). Rare mitotic bodies were seen, and no necrosis was found. Immunohistochemically, the tumor was positive for β-catenin and vimentin, but negative for CD117, SMA, S100, CD34, DOG1, SOX10, and AE1/AE3. Desmin was positive in a few cells, and these features supported the diagnosis of mesenteric fibromatosis.

Postoperative course

Recovery for this patient was uneventful; he remained stable, resumed bowel function, and was discharged on post-operative day 3. Medical oncology evaluation was initiated with molecular testing for FAP, which resulted in a negative for an adenomatous polyposis coli (APC) gene mutation. Given the sporadic nature of mesenteric fibromatosis in this patient, no further treatment was pursued. The role of adjuvant radiotherapy in this case was discussed, but due to the risk of secondary malignancies and unclear benefit of therapy, the decision was made to continue with surveillance alone. Outpatient follow-up care was established with the plan for CT scan surveillance for recurrence every six months.

## Discussion

Mesenteric fibromatosis, also known as desmoid tumor of the mesentery, is a rare fibroproliferative lesion that, despite its benign histology, demonstrates locally aggressive behavior and a high risk of recurrence. These tumors account for less than 0.03% of all neoplasms and approximately 8% of all desmoid tumors, making them an uncommon cause of acute abdominal presentations [8]. The majority of cases occur in younger adults, with a peak incidence between 15 and 60 years, and there is a slight female predominance [2]. Our patient, a 70-year-old male, therefore represents an atypical demographic, further emphasizing the broad clinical spectrum of presentation.

The diagnosis of intra-abdominal desmoid tumors is particularly challenging due to their nonspecific imaging features. On CT, they may appear as well-circumscribed soft tissue masses, but cystic or necrotic variants can mimic intra-abdominal abscesses [10]. This was evident in our case, where the lesion was initially interpreted as a fluid collection consistent with a perforated viscus. Similar diagnostic pitfalls have been highlighted in prior series, where desmoid tumors frequently mimicked malignant or infectious processes, resulting in diagnostic delays [6]. Ultimately, histopathology remains the gold standard for establishing a definitive diagnosis [8,9].

The management of mesenteric fibromatosis remains controversial and must be individualized. Non-operative strategies, including active surveillance, nonsteroidal anti-inflammatory drugs (NSAIDs), anti-estrogen therapy, and tyrosine kinase inhibitors, have been increasingly utilized for asymptomatic or unresectable disease [5]. Surgical resection, once considered the mainstay of treatment, is now typically reserved for patients with symptomatic disease, complications, or when complete resection can be achieved with acceptable morbidity [6]. In our case, urgent laparotomy was indicated due to concern for sepsis, and complete en bloc resection of the tumor with jejunal resection and reconstruction was successfully performed.

Adjuvant therapies such as radiotherapy or systemic treatment were considered but ultimately deferred, as current evidence does not demonstrate a clear benefit in completely resected sporadic cases and may expose patients to unnecessary risks [5]. Close postoperative surveillance with serial imaging is recommended given recurrence rates as high as 30-40% even after margin-negative resections [6].

This case contributes to the growing literature on atypical presentations of mesenteric fibromatosis by demonstrating an unusual occurrence in an elderly male patient, outside the typical age range. It also highlights the potential for radiologic misinterpretation, as the lesion was initially mistaken for a fluid collection, underscoring the limitations of CT in accurately characterizing these tumors. Finally, it emphasizes the need to include mesenteric fibromatosis in the differential diagnosis of atypical intra-abdominal masses, particularly when imaging findings are incongruent with the clinical picture.

A multidisciplinary approach, incorporating surgery, oncology, radiology, and pathology, remains essential to optimize outcomes. This case highlights that, although rare, mesenteric fibromatosis should be considered when imaging findings are discordant with clinical presentation.

## Conclusions

Mesenteric fibromatosis is a rare, benign tumor that can present with deceptive clinical and radiologic features. In this case, an elderly male developed acute abdominal pain with CT findings suggestive of a fluid collection, but intraoperative exploration revealed a mesenteric desmoid tumor. This discrepancy underscores the limitations of imaging alone and the importance of maintaining a broad differential diagnosis when evaluating atypical intra-abdominal findings. Early recognition and multidisciplinary management are essential, and given the high risk of recurrence, close postoperative surveillance remains a critical component of care.
